# Comparative Transcriptome Analysis Reveals the Specific Activation of Defense Pathways Against *Globodera pallida* in Gpa2 Resistant Potato Roots

**DOI:** 10.3389/fpls.2022.909593

**Published:** 2022-06-17

**Authors:** Qi Zheng, André Bertran, Anouk Brand, Casper C. van Schaik, Stefan J. S. van de Ruitenbeek, Geert Smant, Aska Goverse, Mark G. Sterken

**Affiliations:** Laboratory of Nematology, Department of Plant Science, Wageningen University, Wageningen, Netherlands

**Keywords:** *Globodera pallida*, potato roots, R gene, Gpa2, transcriptome, RNAseq, NB-LRR, plant immunity

## Abstract

Cyst nematodes are considered a dominant threat to yield for a wide range of major food crops. Current control strategies are mainly dependent on crop rotation and the use of resistant cultivars. Various crops exhibit single dominant resistance (R) genes that are able to activate effective host-specific resistance to certain cyst nematode species and/or populations. An example is the potato *R* gene *Gpa2*, which confers resistance against the potato cyst nematode (PCN), *Globodera pallida* population D383. Activation of Gpa2 results in a delayed resistance response, which is characterized by a layer of necrotic cells formed around the developing nematode feeding structure. However, knowledge about the Gpa2-induced defense pathways is still lacking. Here, we uncover the transcriptional changes and gene expression network induced upon *Gpa2* activation in potato roots infected with *G. pallida*. To this end, *in vitro*-grown Gpa2-resistant potato roots were infected with the avirulent population D383 and virulent population Rookmaker. Infected root segments were harvested at 3 and 6 dpi and sent for RNA sequencing. Comparative transcriptomics revealed a total of 1,743 differentially expressed genes (DEGs) upon nematode infection, of which 559 DEGs were specifically regulated in response to D383 infection. D383-specific DEGs associated with Gpa2-mediated defense mainly relates to calcium-binding activity, salicylic acid (SA) biosynthesis, and systemic acquired resistance (SAR). These data reveal that cyst nematode resistance in potato roots depends on conserved downstream signaling pathways involved in plant immunity, which are also known to contribute to *R* genes-mediated resistance against other pathogens with different lifestyles.

## Introduction

Potato cyst nematodes (PCN; *Globodera pallida* and *Globodera rostochiensis*) are obligate sedentary plant-parasitic nematodes causing serious damage to potato crops with an estimated yield loss of 9% global production (Jones et al., 2013). To reduce the impact of PCN in crop cultivation, diverse control strategies have been developed. In the past, the use of nematicides helped decrease the impact of nematode infection but had detrimental effects on human health and the soil environment. Therefore, the use of chemical compounds was banned in the early 2000’s (Hillocks, 2012). Later, several biological control strategies emerged, such as the application of bio-fumigation and trap crops (Back et al., 2018). These methods are dependent on the ability of any living organism or biological control agent to reduce the impact of nematode invasion (Back et al., 2018). Biological control often requires natural interactions between effective bio components and cyst nematodes, but the efficiency can be variable (Back et al., 2018). Hence, the most common and sustainable control strategies largely rely on crop rotation with non-host plants and the use of resistant crop cultivars (Gartner et al., 2021). However, the latter may be less efficient over time since resistance-breaking populations can emerge due to genetic host selection (Eoche-Bosy et al., 2017; Mwangi et al., 2019).

In potatoes, several resistance loci are known to neither confer a single dominant nor quantitative resistance to PCN (Varandas et al., 2020; Gartner et al., 2021). Few single dominant resistance (*R*) genes from potatoes have been identified, such as *Gpa2*, *H1*, and *Gro1-4* (van der Vossen et al., 2000; Bakker et al., 2004; Paal et al., 2004). Both *H1* and *Gro1-4* confer resistance to *G. rostochiensis* (Bakker et al., 2004; Paal et al., 2004), while *Gpa2* confers resistance to *G. pallida* (van der Vossen et al., 2000). Compared to *R* genes, an increasing number of resistance trait loci (QTLs) are reported in potatoes. Most QTLs confer resistance to single PCN species, either *G. pallida* (like *Gpa*, *Gpa5*, *Gpa6*, *Pa2/3_A*, and *Pa2/3_B*) or *G. rostochiensis* (like *H1*, *Ro2_A*, and *Ro2_B*) (reviewed by Gartner et al., 2021). However, *Grp1* mediates quantitative resistance to both species (van der Voort et al., 1998; Finkers-Tomczak et al., 2009). QTLs may co-localize with *R* gene clusters within the potato genome, suggesting that *R* genes may underly QTLs-mediated quantitative resistance to PCN (Gartner et al., 2021).

Activation of *R* genes-mediated resistance results in the effective blocking of early steps in the life cycle of PCN leading to a drop in reproduction. Upon penetration of roots, infective second-stage juveniles (J2) move intracellularly to reach the vascular cylinder to establish a permanent feeding site (Varandas et al., 2020). The feeding site develops into the syncytium, a multinucleate structure formed by cell wall dissolution and fusion of neighboring cells. From then on, the parasitic juvenile becomes sedentary and fully relies on the syncytium for the uptake of nutrients and subsequent development into an adult for reproduction. Based on timing in the ontogeny of feeding structures and characteristic cytological features, *R* genes-mediated resistance can be roughly divided into two types (Goverse and Smant, 2014). The first type of resistance is called “male-biased” resistance as induced by, for example, the PCN *R* gene *H1* (Rice et al., 1985). The expansion of the young syncytium is restricted due to a hypersensitive response in neighboring cells and, as such, the syncytium cannot provide sufficient nutrients to support adult female development. Still, sufficient nutrients can be ingested by the nematode to promote the development into males. The second type of resistance occurs in a later stage, as shown in *Gpa2*-mediated resistance. *Gpa2* activation still allows syncytium formation and expansion to initiate female development. However, a layer of necrotic cells formed around the syncytium disconnects the feeding site from the vascular bundle, thereby, developing nematodes cannot acquire sufficient nutrients from the feeding site, leading to starvation and arrest of adult female development (Koropacka, 2010; Goverse and Smant, 2014). This process takes place around 5 to 7 days post-infection (Koropacka, 2010). Despite these histological observations, the signaling pathways and gene expression networks involved in the activation of *R* gene-mediated resistance responses against cyst nematodes are still fragmented.

In this study, we aim to uncover the downstream signaling genes and potential pathways involved in *Gpa2*-mediated resistance during the early stages of PCN infection of potato roots. *Gpa2* is a single dominant *R* gene located on ChrXII in potatoes and encodes an intracellular Coil-Coiled Nucleotide-binding Leucine-rich Repeat (CC-NB-LRR) immune receptor (van der Vossen et al., 2000). We conducted a comparative transcriptome analysis using RNAseq data obtained from infected root segments collected at 3 and 6 dpi from a potato genotype, harboring the Gpa2 gene in its background. *In vitro*-grown potato roots were infected with either the avirulent population D383 or the virulent population Rookmaker, or mock-infected for comparison. We identified a total of 559 genes that were specifically differently regulated upon D383 infection. Those candidate genes most likely contribute to host-specific resistance by *Gpa2* based on their Gene Ontology (GO) and mainly correspond to calcium-binding activity, salicylic acid (SA) biosynthesis, and systemic acquired resistance (SAR). Together, a picture emerges suggesting that SA signaling is likely to be involved in early Gpa2-mediated defense responses to *G. pallida* infection in potato roots. How this compares to other pathways and signaling networks involved in *R* gene-mediated resistance to plant-parasitic nematodes is discussed.

## Materials and Methods

### Plant Material and Potato Cyst Nematode Infection

Nematode infection was performed as described in Goverse et al. (2000). Briefly, stem cuttings of potato genotype SH (carrying Gpa2) were grown *in vitro* in a B5 medium (gradient) at 22°C under a 16-h light/8-h dark cycle. One cutting was grown on each plate. After 2 weeks, each plant was inoculated with approximately 250 surface-sterilized *G. pallida* infective juveniles in roots (Goverse et al., 2000). For the nematode assay, *Globodera pallida* Gpa2-avirulent population D383 and Gpa2-virulent population Rookmaker were used (van der Voort et al., 1997). Inoculation with 0.7% (w/v) Gelrite solution was used as a negative control. Plants were kept at 18°C in the dark and nematode infected potato root segments were collected 3 and 6 days after infection. As the negative control, equal amounts of similar-sized root segments were harvested. The experiment was conducted in three time-separated biological replicates, where each replicate experiment contained all three infection conditions (D383, Rookmaker, or mock) and two time points (3 and 6 dpi).

### RNA Extraction, Library Preparation, and Sequencing

Three biological replicates of 15 plants/samples were collected and snap-frozen in liquid nitrogen. RNA extraction was performed using the Maxwell 16 LEV-plant RNA kit (Promega) following the manufacturer’s protocol. RNA degradation and contamination were monitored on 1% agarose gel. Purification was checked by using the NanoPhotometer^®^ and spectrophotometer (IMPLEN, CA, United States). RNA integrity and quantitation were assessed by using the RNA Nano 6000 Assay Kit of the Bioanalyzer 2100 system (Agilent Technologies, CA, United States). RNA sequencing was done at Novogene (Hong Kong) by using Novaseq 5000 PE150 platform, providing at least 50 million clean paired-end reads of 150 bp per sample. Raw data were deposited at ArrayExpress under E-MTAB- 11646.

### Data Analysis

Except for read mapping, read quantification, and enrichment analysis, all statistical analyses were conducted in R version x64 4.0.2. For data processing and organization, the tidyverse package was used, which included dplyr and ggplot2 (Wickham, 2011; Wickham et al., 2019). The Venn diagram was made with functions from the VennDiagram package. All data figures were generated using R. As all analyses were conducted using custom scripts, we have made these available *via* git: https://git.wur.nl/published_papers/zheng_2022_gpa2.

### RNAseq Data Mapping, Normalization, and Batch Correction

The clean reads were mapped to the reference genome sequence of *Solanum tuberosum* Solgenomics PGSC_v4.03 genome by using HISAT2 software (Kim et al., 2019). Before analysis, the FPKM values were filtered and transformed. First, the potato gene expression was filtered for read detection in all samples. This led to the detection of 19,590 genes out of 39,028 genes in the potato genome. Next, the FPKM values were transformed by


$$F\,P\,K\,M_{l\,o\,g,i,j}=\log_{2}\left(F\,P\,K\,M_{i,j}+1\right)$$

where FPMK_log_ was the log_2_-normalized FPKM value of the gene *I* (one out of 19,590 genes from potato) and sample *j* (one out of 18 samples).

The expression was batch-corrected by first fitting the data to the linear model.


$$F\,P\,K\,M_{l\,o\,g,i,j}=B_{j}+D_{j}+T_{j}+e$$

where FPMK_log_ was the log_2_-normalized FPKM value of the gene *i* (one out of 19,590 genes from potato) and sample *j* (one out of 18 samples), *B* was the experimental batch (1, 2, or 3), *D* was the dpi (3 or 6), and *T* was the treatment (D383, Rookmaker, or mock-infected). For batch correction, the batch-related differences in expression per gene were subtracted from the expression values of the samples within that batch.

For gene-centric analyses [principal component (PCO) analysis and correlation analysis], expression was transformed to a log-ratio with the mean by:


$$F\,P\,K\,M_{r\,a\,t\,i\,o,i,j}=\log_{2}\left(\frac{F\,P\,K\,M_{i,j}}{{\bar{F\,P\,K\,M}}_{i}}\right)$$

where the mean FPKM_ratio_ was the log_2_ of the FPKM value of gene i (one out of 19,590 for potato) and sample j (one out of 18 samples for potato), divided by the average FPKM value over all samples for gene i.

### Principal Component Analysis

To understand the factors causing the variance in the expression data, PCO analysis was performed with the *prcomp* function in R with the parameter *scale*. = *TRUE* on the FPKM_ratio_-transformed expression data.

### RNAseq Liner Model and Differentially Expressed Genes

To identify differentially expressed genes (DEGs) associated with nematode infection, a linear model was applied:


$$F\,P\,K\,M_{l\,o\,g,i}=G_{D\,383,j}+G_{R\,o\,o\,k,j}$$

where the gene-expression FPKM_log_ of gene *i* was explained over the genotype being D383 (*G_*D*383_*) or Rookmaker (G_Rook_) of sample *j*. This model was applied for 3 dpi and 6 dpi separately.

We selected DEGs based on a threshold of *p* < 0.001. This was at a false discovery rate (FDR) adjustment for multiple testing (provided by the *p.adjust* function) of.037 for D383 at 3dpi,0.043 for Rookmaker at 3dpi,0.017 for D383 at 6dpi, and 0.020 for Rookmaker at 6 dpi.

### Venn Diagram and k-Means Clustering

To visualize the overlap of significantly DEGs (*p* < 0.001) in D383 and Rookmaker infected groups at 3 and 6 dpi Venn-diagrams were used.

To visualize expression patterns of significantly DEGs, k-means clustering was performed to divide genes into several clusters with similar expression patterns. In the end, 1–20 clusters were examined using kclust and 5 clusters were selected for visualizing the data. The final analysis was run with the parameters iter.max = 1,000 and nstart = 50.

### Gene Ontology Term Enrichment Analysis

The GO term enrichment analysis was done by the online tool g: Profiler.^1^

### Reverse-Transcriptase Quantitative PCR Analysis

Total RNA extracted from the infected root segments of samples was used for cDNA synthesis and evaluation of gene expression by RT-qPCR. For reverse transcriptase, complementary DNA (cDNA) was synthesized with 400 ng of total RNA using the GoScript™ reverse transcriptase and Oligo-(dT)_12–18_ (Invitrogen). To analyze expression levels, RT-qPCR was done (BioRad System) in a total reaction mix of 10 μl consisting of 0.5 μl forward and reverse primers (0.5 μM each), 5 μl IQ™ supermix, 1 μl MQ water, and 3 μl cDNA. RT-qPCR was run using the following program: initial denaturation at 95°C for 3 min followed by 40 cycles of amplification at 95°C for 15 s, 59.4°C for the 30°s, and 72°C for 15°s. After amplification, melt curve analysis was run from 65 to 95°C with every 5 s and 0.5°C increment. Each biological sample was analyzed *in triplo*. A standard no template control was included to indicate the presence of contaminating DNA. Four housekeeping genes RPN7, MST2, OXA1 (Miranda et al., 2013), and TUA5 (Castro-Quezada et al., 2013) were used for RT-qPCR data normalization. Target genes were normalized based on reference genes with the closest Ct values. This means that the data is comparable between samples, but not between different target genes. All primer sequences used were summarized in Supplementary Table 1. Finally, relative expression levels were analyzed by the comparative method (2^–ΔΔ^ ^Ct^) using the average threshold values. Expression values (calculated by CFX Maestro) were used as input for data visualization in R-studio version 2022.02.0, using R package ggplot2 (Wickham, 2016). Statistical differences between gene expression of treatment groups and the control group were measured using a two-sample unpaired *t*-test followed by one-way ANOVA and Tukey’s HSD pairwise comparison.

## Results

### Differential Expression Analysis Reveals Genes Sets Linked to *Gpa2*-Mediated Resistance

**FIGURE 1:**
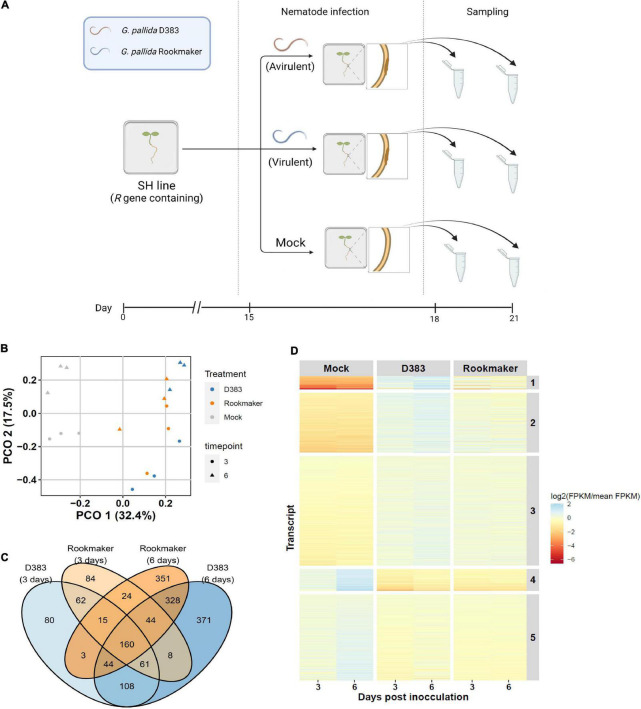
Overview of *Globodera pallida* infection on potato roots. **(A)** Segments of Gpa2-containing potato genotype SH were explanted in B5 medium and (mock-)infected with *G. pallida*. At 15 dpi, we applied Gpa2-avirulent population D383 (orange), Gpa2-virulent population Rookmaker (blue), or Gelrite (Mock). The root segments infected with *G. pallida* were harvested at 3 and 6 dpi for RNA sequencing. For the non-infected control (Mock), similar-sized root segments were collected randomly. This figure was created with biorender.com. **(B)** The first two Principal components (PCO) of gene expression in potato roots. The first PCO explains 32.4% of the variance and is associated with nematode infection. The second PCO explains 17.5% of the variance and is associated with time after nematode infection. The samples at 3 and 6 dpi are shown as dots and triangles, respectively. Colors indicate samples infected with nematodes (D383 population, blue; Rookmaker population, orange) and without nematodes (Mock, gray). **(C)** Venn diagram showing the overlap in significantly differentially expressed DEGs in *G. pallida* infected potato roots. In total 1,743 DEGs (*p* < 0.001, FDR < 0.05) are involved in plant-nematode interactions. **(D)** Heat map of k-means clustering of 1,743 significantly differentially expressed genes over all samples. Differentially expressed genes in different treatments at both 3 and 6 dpi can be divided into five clusters. The color scale indicates the median expression over three replicates (red, lower expression; blue, higher expression).

To investigate the transcriptional responses induced by the resistance gene *Gpa2* upon *G. pallida* infection, we performed an *in vitro* nematode infection assay on the diploid, Gpa2 containing potato genotype SH (Figure 1A). We used both the Gpa2-virulent population E400 Rookmaker and the Gpa2-avirulent population D383 of *G. pallida* (van der Voort et al., 1997). As an uninfected control, we mock-inoculated potato roots with Gelrite, a solution in which the infective nematode juveniles were suspended for homogeneous infection of the treated roots (Mock). To capture the *Gpa2*-mediated early and local responses, nematode-infected root segments were harvested at both 3 and 6 dpi, and RNA was extracted and sent for sequencing. These time points were chosen because the earliest necrosis around the syncytia was observed for D383 around 7 dpi (Koropacka, 2010), suggesting that Gpa2 initiates resistance response pathways earlier than 7 dpi. For each treatment, we included three biological repeats, resulting in 18 samples in total. After quality filtering, each sample showed high-quality sequences [Q30 (%)] ranging from 92.7 ∼ to 94.6% (Supplementary Table 2) and could be used for reads mapping. The total reads mapping rate of each sample was around 78.7 ∼ 87.8% (Supplementary Table 2). Thereafter, mapping results were used as inputs for further bioinformatics analysis.

To investigate which factors affect the variance in the messenger RNA (mRNA) abundance in potatoes, we performed a PCO analysis. The major PCO1 represented 32.4% of the variance and was connected with nematode infection and the second PCO2 represented 17.5% of the variance and was associated with time (Figure 1B). Taken together, infection and time were the dominant contributors to variance over the samples. Next, to investigate which genes were affected because of nematode infection and time, we determined the gene expression changes using a linear model. We compared the expression level of genes from D383-treated groups with Mock groups and a similar comparison was done for Rookmaker as well. Analysis showed that in total, 1,743 genes were significantly DEGs during nematode infection (*p* < 0.001, FDR < 0.05) (Supplementary Figure 1 and Supplementary Table 3).

Subsequently, the overlap between the timespoints and treatments was determined (Figure 1C and Supplementary Table 4). D383-specific DEGs were thought to include DEGs in response to the incompatible plant-nematode interactions between the avirulent *G. pallida* population’s D383 and *Gpa2*-resistant potato roots. Among the 559 D383-specific DEGs, 371 DEGs were found to be specifically and differentially expressed at 6 dpi, 108 DEGs at both 3 and 6 dpi, and 80 DEGs were specific for 3 dpi (Figure 1C). Rookmaker-specific DEGs include genes that are presumed to be associated with the compatible plant-nematode interaction as this virulent population can establish a normal feeding relationship and does not trigger Gpa2 resistance in potato roots (van der Voort et al., 1997). Among the 459 Rookmaker-specific DEGs, 351 DEGs were found to be specifically and differentially expressed at 6 dpi, 84 at 3 dpi, and 24 at both 3 and 6 dpi (Figure 1C). These specific sets of Rookmaker DEGs formed a reference set to identify specific DEGs for the transcriptional regulation of genes during Gpa*2*-mediated immunity against D383.

To group and identify expression patterns of DEGs over time, we used k-means clustering of the 1,743 DEGs for all samples. We found that the expression patterns of the DEGs could be well-explained by five clusters with similar expression patterns (Figure 1D and Supplementary Table 5). DEGs from cluster 1 (80 DEGs), cluster 2 (359 DEGs), and cluster 3 (657 DEGs) showed upregulation after nematode infection at both 3 and 6 dpi, where DEGs from cluster 1 and 2 showed the highest upregulation after nematode infection, especially in D383 at 6 dpi. In contrast, DEGs from cluster 4 (128 DEGs) and cluster 5 (519 DEGs) showed downregulation after nematode infection as time progressed. Especially, DEGs in cluster 4 showed downregulation after nematode infection at both 3 and 6 dpi. Overall, especially, D383 infection was strongly associated with upregulation of gene expression when compared to Rookmaker infection. From this, we concluded that D383 infection results in activation of gene expression linked to the activation of host-specific *Gpa2*-mediated defense responses by the D383 population.

### Identification of Specific Differentially Expressed Genes in Response to D383 Population Infection

**FIGURE 2:**
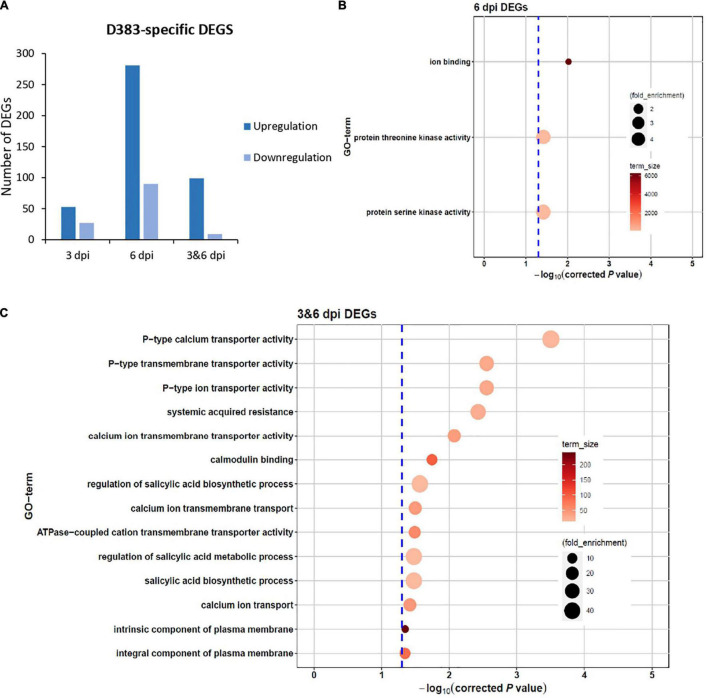
Differentially expressed genes (DEGs) specific for Gpa2-mediated resistance in potato roots infected with the avirulent *Globodera pallida* population D383. **(A)** Overview of the total number of up- and down-regulated DEGs at 3 dpi, 6 dpi or both. No results for 3 dpi under the settled threshold of 0.05. GO-term analysis on D383-specific DEGs at **(B)** 6 dpi or **(C)** both 3 and 6 dpi.

To investigate the DEGs that specifically respond to Gpa2 activation, we focused on D383-specific DEGs for further analysis. In response to D383 infection, a total of 433 and 126 DEGs showed up- and down-regulation, respectively (Figure 2A). Among the upregulated DEGs, 53 and 281 were from 3 and 6 dpi, respectively, and 99 DEGs were from both 3 and 6 dpi (Figure 2A). For the downregulated DEGs, 27 and 90 were from 3 and 6 dpi, respectively, and 9 DEGs were from both 3 and 6 dpi (Figure 2A). Regardless of whether D383-specific DEGs were up- or down-regulated, the number of DEGs from 3 dpi was much less than 6 dpi, suggesting that more DEGs could be detected in the *Gpa2*-mediated response as time developed.

To better understand the function of those highly regulated D383-specific DEGs, we performed a GO term enrichment analysis using the gProfiler online tool based on different time points, regardless of whether these DEGs were up- or down-regulated. Unfortunately, there was no enrichment result for 80 DEGs at 3 dpi (FDR = 0.05). It is possible that at this time point in the immune response, only a few specific genes were activated, and, therefore, no specific processes were found to be enriched. The 371 DEGs at 6 dpi were mainly involved in ion binding and threonine/serine kinase activities (Figure 2B), which are important for plant-pathogen interactions (Afzal et al., 2008; Aldon et al., 2018). For 108 DEGs enriched at both 3 and 6 dpi, data showed that they were mainly involved in SAR, SA biosynthetic process, calmodulin-binding, and multiple transporter activities, such as calcium, ion, and transmembrane transporter (Figure 2C). These GO terms show clear potential links to downstream defense responses, triggering our interest to have a closer look at the specific individual DEGs from these categories. The SAR category (GO:0009627) mainly consisted of genes encoding an NPR1 interacting protein, Aminotransferase ALD1, an ortholog of Arabidopsis Lipase-like PAD4, and an unknown protein (Table 1). Genes involved in the regulation of the SA biosynthetic process (GO:0080142) mainly encoded Calmodulin-binding proteins and an ortholog of Arabidopsis Lipase-like PAD4 (Table 1). Genes in the calcium ion transmembrane transport category (GO:0070588) encode diverse Calcium-transporting ATPases (Table 1).

**TABLE 1:** Gene Ontology (GO)-term enrichment analysis (FDR = 0.05) of downstream defense response-related D383-specific differentially expressed genes (DEGs) at both 3 and 6 dpi.

Term_name	Term_id	DEGs	Short annotation
Systemic acquired resistance	GO:0009627	PGSC0003DMG400014558	NPR1 interacting
		PGSC0003DMG400005762	–
		PGSC0003DMG400022929	Aminotransferase ALD1
		PGSC0003DMG400019873	Ortholog of Arabidopsis Lipase-like PAD4
Regulation of salicylic acid biosynthetic process	GO:0080142	PGSC0003DMG400024785	Calmodulin-binding protein 60 B
		PGSC0003DMG400024478	Calmodulin binding protein-like
		PGSC0003DMG400019873	Ortholog of Arabidopsis Lipase-like PAD4
Calcium ion transmembrane transport	GO:0070588	PGSC0003DMG400021421	Calcium-transporting ATPase 2
		PGSC0003DMG400019431	Putative calcium-transporting ATPase 13
		PGSC0003DMG400013022	Calcium-transporting ATPase 2
		PGSC0003DMG400018942	Calcium-transporting ATPase 12

**FIGURE 3:**
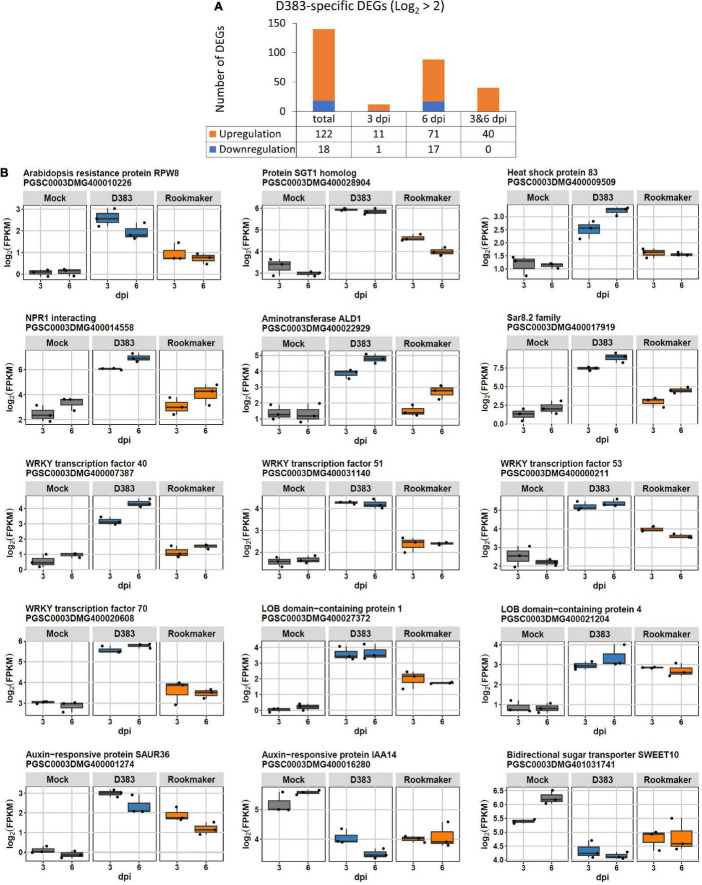
Differentially expressed genes (DEGs) that are highly involved in Gpa2-mediated resistance (log_2_ > 2). **(A)** Overview of the numbers of highly regulated DEGs at 3 dpi, 6 dpi or both. **(B)** Expression level of representative D383-specific DEGs (log_2_ > 2) that relate to defense response or nematode feeding site development.

To further identify DEGs that are highly associated with Gpa2-mediated defense responses, we selected D383-specific DEGs with a large effect size (log_2_ > 2). This selection contained 122 up- and 18 down-regulated DEGs (Figure 3A). In line with the k-means clustering (Figure 1D), the 122 upregulated DEGs were from either cluster 1 or 2, while the 18 downregulated DEGs were all from cluster 4 (Supplementary Table 6). Among these 122 upregulated DEGs, 11 and 71 DEGs were specifically upregulated at 3 and 6 dpi, respectively (Figure 3A). Forty DEGs were upregulated at both 3 and 6 dpi, with most of them showing a comparable or slightly increased expression level over time (Figure 3A). The upregulated DEGs induced at both 3 and 6 dpi included genes encoding: a Sar8.2 family protein, WRKY transcription factors, and NPR1 interacting protein, LOB domain-containing proteins, an auxin-responsive protein, an SGT1 homolog, the Aminotransferase ALD1, an ethylene-responsive transcription factor, and a receptor-like protein kinase (Supplementary Table 6). The DEGs that were specifically upregulated at 3 dpi included genes encoding as follows: a tyramine N-feruloyl transferase, an ortholog of Arabidopsis broad-spectrum mildew resistance protein RPW8, and cytochrome P450 (Supplementary Table 5). DEGs that are only upregulated at 6 dpi mainly encoded the following: lipid transfer proteins, a germin-like protein, pathogenesis-related protein, WRKY transcription factors, a calcium-binding protein or calcium-transporting ATPases, receptor-like serine/threonine-protein kinases, two cytochrome P450, a Myb-related protein, and a heat shock protein (HSP) (Supplementary Table 6).

Different from the upregulated DEGs, there were no DEGs detected that were downregulated at both 3 and 6 dpi under the conditions used. Among 18 downregulated DEGs, 1 and 17 were exclusively downregulated at 3 and 6 dpi, respectively (Figure 3A). The only downregulated transcript at 3 dpi encodes a plastocyanin-like domain protein (Supplementary Table 6). The DEGs exclusively downregulated at 6 dpi included genes encoding abscisic acid and environmental stress-inducible protein, a cytochrome P450, peroxidases, bidirectional sugar transporters, an auxin-responsive protein, ethylene-responsive transcription factor, and a MAPK/ERK kinase (Supplementary Table 6). In general, most upregulated DEGs were associated with plant defense responses, whereas several downregulated DEGs were mostly involved in other hormone-regulated activities (see representative DEGs in Figure 3B and Supplementary Table 6).

### Reverse-Transcriptase Quantitative PCR Validation of Differentially Expressed Genes Obtained From the RNAseq Analysis

To validate the DEGs from the RNAseq data, we selected 12 genes for assessment of their transcript levels using RT-qPCR. For both 3 and 6 dpi, we selected 3 genes that showed up- and down-regulation, respectively. These genes, including some representative DEGs shown in Figure 3B (WRKY40/51/70, IAA14, and SWEET10), were functionally associated with plant defense response to nematodes and showed varying expression levels (log_2_ range from 0.5 to > 3). Results showed that these genes indeed present different expression patterns and that these patterns were highly similar to the expression patterns obtained by RNAseq analysis (Supplementary Figure 2). From this, we conclude that the results from RNAseq and RT-qPCR analysis were consistent and that the RNAseq results were robust over a wide range of log_2_ fold changes. 0

## Discussion

In this study, we performed RNA sequencing of *in vitro* Gpa2-resistant potato roots infected with the cyst nematode *G. pallida* using the avirulent population D383 and the virulent population Rookmaker. Root segments were collected at 3 and 6 dpi to investigate the genes differentially expressed upon specific activation of the Gpa2 resistance gene. Comparative transcriptome analysis between D383 and Rookmaker-infected root segments obtained from the same genetic potato background showed a total of 1,743 genes differentially expressed upon nematode infection, of which a subset of 559 genes was specifically regulated upon Gpa2 activation in response to D383 infection. GO enrichment analysis showed that D383-specific DEGs were mainly involved in downstream defense responses, such as calcium ion transmembrane transport, SA biosynthetic process, and SAR. Closer inspection of highly upregulated DEGs (log_2_ > 2) was associated with plant defense activities, whereas highly downregulated DEGs were mainly associated with other hormone-regulated activities. These data reveal candidate genes and potential pathways involved in Gpa2-mediated defense responses to cyst nematode infection in potato roots.

### Genes Potentially Involved in Maintaining Steady Levels of the Gpa2 Protein in Plant Cells

The stability of NLRs is maintained by molecular chaperones including SGT1, HSP90, and RAR1 (Kadota et al., 2010). Therefore, it is likely that also Gpa2 requires such co-chaperones to maintain protein stability and a steady-state pool in plant cells pre-activation, enabling a rapid defense response upon nematode infection. Indeed, there were two highly upregulated (log_2_ > 2) D383-specific DEGs encoding an SGT1 homolog and a HSP 83, of which the latter is a member of the HSP90 family. Their enhanced expression level may indicate that SGT1 and HSP90 also function post-activation, probably by stabilizing *de novo*-synthesized Gpa2 immune receptors or other components of the resistance complex to mediate a proper and efficient immune response upon nematode detection. Alternatively, SGT1 and HSP90 could play other roles in the downstream activation of nematode defense responses. For Rx1, a close homolog of Gpa2, SGT1, for example, was shown to play a role in protein stability (Botër et al., 2007), but also in the nucleocytoplasmic distribution of Rx1 in the cell in a phosphorylation-dependent manner (Hoser et al., 2014). Moreover, HSP90 is reported to be required for defense activation for some NLRs, including RPS4, Pto, N, and Rx1 (Lu et al., 2003; Zhang et al., 2004). Therefore, it will be interesting to investigate the role of SGT1 and HSP83 in Gpa2 functioning to see how this links to nucleocytoplasmic dynamics and/or changes in protein stability.

### Salicylic Acid-Dependent Signaling Is Likely Involved in Gpa2-Mediated Resistance

Differential gene expression upon Gpa2-mediated defense responses points to a prominent role for salicylic acid (SA) in host-specific resistance to PCN. D383-specific DEGs showed that NLR signaling components PAD4, EDS1, and NDR1 were upregulated at both 3 and 6 dpi, and upregulation was slightly increased over time (Supplementary Figure 3). Commonly, PAD4/EDS1 is required for TNLs and certain CNLs, like Arabidopsis RPS2 and HRT (Venugopal et al., 2009; Cui et al., 2017), whereas NDR1 is required for most CNLs to activate downstream defense signaling (van Wersch et al., 2020). PAD4/EDS1 and NDR1 are two different signaling branches for NLRs, but they both function upstream of SA and positively enhance SA accumulation (Shapiro and Zhang, 2001; Cui et al., 2017; Sun et al., 2021). From this, we propose that the EDS1/PAD4 and NDR1 branches might be involved in the activation of SA-mediated defense pathways in Gpa2-mediated resistance to cyst nematodes in potato roots. The downstream signaling of SA-mediated defense is predominantly regulated by NPR1, a master regulator of the SA pathway, and SA-induced SAR (Backer et al., 2019). SAR is a long-distance signaling mechanism that confers long-lasting resistance against secondary infections in plants (Gao et al., 2015). Recently, NPR1 has been shown to interact with EDS1, but not PAD4, to upregulate EDS1 at the transcriptional level. Vice versa, it was reported that EDS1 stabilizes NPR1 at the protein level (Chen et al., 2021). Interestingly, an NPR1 interacting protein was highly upregulated at both 3 and 6 dpi upon D383 infection of Gpa2 resistant plants (Figure 3B). Although NPR1 was not detected in our data set, the upregulation of this NPR1-interacting protein may provide circumstantial evidence that NPR1, as a key regulator of SA-mediated defense, contributes to Gpa2 immunity to cyst nematodes together with other components like EDS1. However, further research is needed to test these hypotheses.

Functionally, NPR1 associates with TGACG-binding (TGA) and WRKY transcription factors (TFs) in the nucleus to modulate SA-mediated downstream signaling during defense responses (Backer et al., 2019). In this study, WRKYs emerged as a major group of TFs showing differential expression in response to D383 infection (Supplementary Table 3), further supporting a potential role for NPR1 in Gpa2-mediated plant immunity. Particularly, *WRKY40*/*51*/*53*/*70* were highly upregulated (log_2_ > 2) at both 3 and 6 dpi (Figure 3B). Two of these WRKYs have been previously found to be involved in SA-dependent defense responses. First, *WRKY40* gene expression is induced by *Pseudomonas syringae* DC3000 effector AvrRPS4-triggered defense response (Schön et al., 2013). Second, *WRKY70* is required for CNL *Mi-1*-mediated resistance against root-knot nematode *Meloidogyne javanica* in tomatoes (Atamian et al., 2012). *WRKY53* has a functionally redundant role with *WRKY70* in defense signaling induction and contributes to basal defense (Wang et al., 2006; Murray et al., 2007). However, upon Gpa2 activation, both *WRKY53* and *WRKY70* were upregulated, suggesting *WRKY53*/*70* may function cooperatively in NLR-mediated defense responses. In addition, *WRKY70*/*51* has an antagonistic effect on the SA and Jasmonic acid (JA) pathways and positively regulates the SA response (Zhang et al., 2018). Overall, upregulation of SA-mediated defense response regulators *WRKY40*/*51*/*53*/*70* (Caarls et al., 2015) underscores that the SA defense pathway is likely involved in Gpa2-mediated defense response.

The co-regulation of NPR1 and TGA/WRKY TFs often results in an enhanced expression of SA-responsive genes, like AGD2-like defense response protein 1 (ALD1), which was highly upregulated (log_2_ > 2) at both 3 and 6 dpi and grouped in the SAR GO-term category (GO:0009627) (Table 1 and Figure 3B). Similarly, ALD1 has been shown to improve soybean resistance to soybean cyst nematode *H. glycines* (Kang et al., 2018). ALD1 carries aminotransferase activity, which is essential for the biosynthesis of SAR-inducer Pip, to initiate a distal defense response known as SA-induced SAR (Gao et al., 2015). In the present study, we think ALD1 may positively regulate systemic defense levels in the potato root system or even in the aboveground plant parts upon local Gpa2 activation upon cyst nematode infection. This hypothesis is further supported by the notice that the gene encoding Sar8.2 family protein shows the highest upregulation in D383-specific DEGs at both 3 and 6 dpi (Figure 3B). The Sar8.2 family is involved in SA-induced SAR (Song and Goodman, 2002) and we speculated that the Sar8.2 family protein may function downstream of ALD1 in Gpa2-mediated defense responses. It will be interesting to see if, indeed, hallmark genes of SAR are upregulated in distal plant tissues and how this may increase plant defense levels against other pathogens below- and aboveground.

### Calcium Signaling Seems Associated With Gpa2-Mediated Resistance

Calcium signaling appears to be a major component in downstream signaling induced upon Gpa2-mediated resistance. We found 22 D383-specific DEGs encoding proteins that are involved in calcium signaling activity, including calcium-binding proteins, calcium exchanger proteins, calcium uniporter proteins, and calcium transporting ATPase (Supplementary Table 4). Moreover, some of these were highly upregulated (log_2_ > 2) at 6 dpi (Supplementary Table 6). This is consistent with GO enrichment analysis that D383-specific DEGs, enriched at both 3 and 6 dpi, are associated with calcium ion transmembrane transport (GO:0070588) (Table 1). Those calcium-activity-related proteins may contribute to calcium signaling activity in the cells upon Gpa2-mediated immune responses. Calcium flux is important in initiating both basal defense and *R* genes-mediated resistance in plant cells and the blocking of calcium signals largely impairs plant immune response (Aldon et al., 2018). Different from a transient calcium signal generated in basal defense, a long-lasting cytosolic calcium signal is detected during *R* gene-mediated resistance (Aldon et al., 2018). The activated NLR ZAR1 resists some forms of a calcium influx channel to activate subsequent HR and immune responses (Bi et al., 2021). Therefore, the enrichment of those calcium signaling activity-related genes suggests there might be increased calcium influx upon defense activation, leading to Gpa2-mediated cell death around the nematode feeding sites (Koropacka, 2010).

### Other Extra- and Intracellular Immune Co-receptors May Contribute to Gpa2-Induced Resistance

Several known immune receptors show significant upregulation in response to D383 infection, indicating that they might be part of the Gpa2-mediated resistance response. First, upregulation of an ortholog of AtRPW8 was detected at 3 dpi (Figure 3B). AtRPW8 is an atypical intracellular immune receptor containing a CC domain that shares sequence homology to the CC domain of the third type of NLRs named RPW8-NLRs, which function as helper NLRs to mediate downstream signaling for multiple sensor NLRs from both CNL and TLN types (Castel et al., 2019). AtRPW8 confers broad-spectrum resistance to mildew but also enhances resistance against diverse pathogens in both Arabidopsis and rice by boosting PTI signaling (Li et al., 2018). The regulation of AtRPW8 occurs *via* an SA-dependent pathway, which requires EDS1 and PAD4 for function (Xiao et al., 2001, 2003, 2005). Moreover, transcription of RPW8 is controlled in an SA-dependent feedback loop. However, there is no direct evidence yet to show that RPW8 is involved in NLRs-mediated immunity. Therefore, we speculate that the enhanced expression of RPW8 is caused by enhanced SA signaling during Gpa2 activation. An alternative hypothesis is that this RPW8 homolog functions as a helper NLR in Gpa2 mediated immunity to cyst nematodes in potatoes in an SA-dependent manner as demonstrated for other sensor NLRs (Castel et al., 2019).

Additionally, receptor-like protein kinases (RLKs) were also upregulated in response to D383 infection, including receptor-like tyrosine-protein kinase FERONIA and leucine-rich repeat receptor-like serine/threonine/tyrosine-protein kinase SOBIR1 (Supplementary Figure 3). RLKs locate in the plasma membrane and they perceive pathogens through their extracellular domain and initiate the defense signal through their intracellular domain (van der Burgh and Joosten, 2019). SOBIR1 may probably enhance Gpa2-mediated (systemic) resistance *via* SA-dependent signaling as it can directly associate receptor-like proteins with EDS1 and PAD4, which are considered the core signaling node for both PTI and ETI (Pruitt et al., 2020). In addition, SOBIR1 shows elevated phosphorylation levels in SAR-induced Arabidopsis leaves, suggesting SOBIR1 is likely involved in SAR in a phosphorylation-dependent regulation (Zhou et al., 2021). As SAR seems involved in Gpa2-mediated resistance as shown in GO enrichment analysis (Figure 2C), we think the upregulation of SOBIR1 at 6 dpi may partly contribute to signaling communication between neighboring cells in Gpa2-mediated SAR. FERONIA has been shown to potentiate defense response to *Pseudomonas syringae* DC3000 *via* suppression of JA signaling in Arabidopsis (Guo et al., 2018). This may also occur during Gpa2 activation since JA can antagonize SA signaling and SA appears to dominate Gpa2-mediated downstream resistance. Alternatively, these findings may point to the cross-talk between effector-triggered immunity upon Gpa2 activation and pathways involved in PTI as recently described (Yuan et al., 2021).

### The Development of Nematode Feeding Sites Is Arrested Upon Gpa2 Activation

The activation of Gpa2 leads to the arrest of cyst nematode feeding site development. Auxin is known to be crucial for plant root formation and nematode feeding site development and consists of three major classes: IAA, GRETCHEN HAGEN 3 (GH3), and small auxin up RNA (SAUR) (Oosterbeek et al., 2021). We found downregulation of IAA14 at 6 dpi; this gene is particularly important for lateral root formation and is induced during feeding site establishment (Fukaki et al., 2002; Grunewald et al., 2008). Similarly, gene expression of the SAUR family is often detected in nematode feeding sites, and the majority of SAUR genes is downregulated during nematode infection (Oosterbeek et al., 2021). SAURs can modulate IAA transport and, thereby, affect the cell expansion and division process (Ren and Gray, 2015). In this study, we detected an opposite response by the upregulation of SAUR36 at both 3 and 6 dpi upon Gpa2 activation (Figure 3B). There are two possible explanations. First, SAUR36 upregulation might prevent further expansion of the feeding sites, or second, SAUR36 upregulation could also be a passive secondary response due to feeding cell collapse upon Gpa2 immunity. In the latter case, we consider that Gpa2-mediated cell death is initiated earlier than 5 dpi.

Similarly, several other genes were found that may have a suppressive effect on nematode feeding site development. For example, two LOB domain-containing proteins (LBD1 and LBD4) showed high upregulation in response to D383 population infection (Figure 3B). LBD16 is characterized as a key component of the auxin pathway to participate in cell divisions during lateral root formation (Cabrera et al., 2014). LBD16 is upregulated during root-knot nematode *M. javanica*-induced gall formation in Arabidopsis but for cyst nematode *H. schachtii*-induced syncytia, in which LBD16 expression level is not affected (Cabrera et al., 2014). Instead, LBD16 is downregulated in transcriptional data from *H. schachtii*-induced syncytia (Szakasits et al., 2009). Considering LBD1 and LBD4 belong to the same protein family of LBD16, we speculate that LBD1 and LBD4 might be involved in Gpa2-mediated resistance *via* suppression of syncytium formation and lateral root formation.

Sugar transporters play an important role during plant-nematode interactions (Hofmann and Grundler, 2007; Hofmann et al., 2009; Zhao et al., 2018). During the syncytium formation, the amount of the soluble sugar should be sufficient for cyst nematode development as the syncytium is the only nutrient source. A previous study has shown various sugar transporters are specifically induced in syncytium in *H. schachtii* infected Arabidopsis (Hofmann et al., 2009). This study showed a significant downregulation at 6 dpi of sugar transporter, Sugars Will Eventually Be Exported Transporters 10 (SWEET10) (Figure 3B). SWEET10 can enhance the resistance to the fungus *Fusarium oxysporum* in sweet potatoes (Li et al., 2017). Meanwhile, the expression level of SWEET10 is increased in tomatoes in response to root-knot nematode *M. incognita* infection (Zhao et al., 2018), demonstrating a potential role during nematode infection. Altogether, we think SWEET10 might contribute to Gpa2-mediated resistance *via* disruption of sugar transport for nematode development.

### Downstream Signaling Involved in *R* Genes-Mediated Resistance to Potato Cyst Nematodes

In recent years, several transcriptomic analyses have been done to study the downstream signaling pathways involved in *R* genes-mediated resistance against PCN. For instance, a transcriptome study on resistant and susceptible tomato roots infected with PCN *G. rostochiensis* indicates the SA pathway plays a vital role in *Hero A*-mediated resistance. Upon PCN infection, SA hallmark gene *PR1* shows enhanced expression level in resistant tomato roots while insertion of *NahG*, which can reduce endogenous SA in plants, in *Hero A*-containing resistant tomato line compromises the upregulation of *PR1* (Uehara et al., 2010). Moreover, PCN produces a higher number of cysts in *Hero A-NahG* plants compared to *Hero A*-containing plants, demonstrating that SA directly controls the *Hero A*-mediated resistance (Uehara et al., 2010). Likewise, transcriptome analysis is also performed in resistant and susceptible potato roots infected by PCN *G. rostochiensis* and resistant lines containing the *H1* gene in the background (Walter et al., 2018). Resistance activation leads to the upregulation of several genes including germins, a stress-responsive factor TSRF1 (tomato stress-responsive factor), a cysteine protease, and a laccase (Walter et al., 2018). Resistant plants show the upregulation of TSRF1, which encodes an ERF-type transcription factor, suggesting ethylene might be involved in *H1*-mediated resistance (Walter et al., 2018). In addition, the downregulation of a Myb R2R3 homology in resistant plants suggests JA might be suppressed in response to PCN (Walter et al., 2018).

Similar to these transcriptome studies, our study also indicates that SA/JA/ET is involved in Gpa2-mediated resistance to PCN; in particular, SA plays a vital role. Besides, we imply ABA has a potential role in Gpa2 functioning. This evidence is in line with the well-reported role of the above signaling in plant defense responses against various pathogens (Koornneef and Pieterse, 2008; Ali et al., 2018; Przybylska and Obrępalska-Stęplowska, 2020; Sun and Zhang, 2021); however, different from *H1/Hero A* transcriptome studies, which are all based on data comparison between the resistant and susceptible cultivars. We only infected resistant potato roots with a Gpa2-virulent population and an avirulent population of *G. pallida*. Moreover, we collected local infected root segments instead of the whole root system. Although Hero A, H1, and Gpa2 all result in a layer of necrotic cells around the feeding sites, Hero A and H1 activate resistance in an early stage, while Gpa2 leads to delayed resistance (Goverse and Smant, 2014). We noticed that in the Hero A study, samples are harvested at a very late stage (90 dpi) (Uehara et al., 2010), while in the H1 study, samples are harvested at an early stage (8 and 48 dpi) (Walter et al., 2018). The different experimental designs (plant genotypes, nematode pathotypes, and harvest timespoints) could be major factors resulting in different results from individual studies. Compared to the Hero A and H1 studies, results from our Gpa2 study display a picture that focuses on plant root local defense responses against PCN and here we removed the plant genotype background noise. However, we cannot exclude that different nematode species or populations may also release different molecular components in plant cells causing the subsequent regulation of other sets of genes.

**FIGURE 4:**
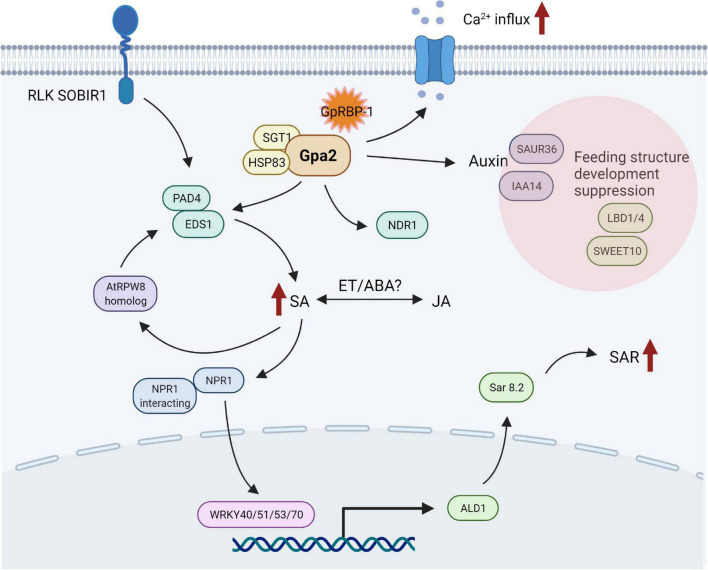
Overview of candidate genes and pathways potentially involved in Gpa2-mediated resistance in potato roots infected with the cyst nematode *Globodera pallida*. Referring to available functional studies of candidate genes, a picture emerges in which the activation of Gpa2 leads to association with SGT1/HSP83 to stabilize Gpa2 in the cytoplasm. Next, Gpa2 might recruit EDS1/PAD4 to upregulate salicylic acid (SA) signaling and activate subsequent SAR in neighboring cells. Receptor like kinase SOBIR1 and resistance protein AtRPW8 homolog seem to contribute to resistance *via* SA signaling. Jasmonic acid (JA), ethylene (ET), and abscisic acid (ABA) might be involved in Gpa2 activation *via* crosstalk with SA. Downregulation of auxin-responsive genes, together with LBD1/4 and SWEET10, is probably associated with suppression of nematode feeding site development upon activation of Gpa2-mediated defense responses. The Ca^2+^-related activities positively regulate the Gpa2-mediated hypersensitive response in the cells. This figure was created with biorender.com.

In conclusion, Gpa2 activation upon *G. pallida* infection results in the differential expression of genes related to transcription factors, phytohormones, and calcium-binding proteins. These responses are already initiated at 3 dpi and are amplified at 6 dpi. Our data suggest that SA signaling seems to be involved in Gpa2-mediated resistance to D383 infection. Other phytohormones, such as JA/ethylene/SA, may also be involved in Gpa2 activation. Meanwhile, activation of Gpa2 is likely to suppress nematode feeding site development. Together, these activities result in the eventual calcium-dependent and Gpa2-mediated delayed cell death response around the feeding sites. Together, we propose a general first picture of downstream defense responses induced by the Gpa2 immune receptor upon recognition of the avirulent *G. pallida* population D383 in potato roots (Figure 4). Furthermore, our data imply that there might be a trade-off between plant defense responses against cyst nematodes on the one hand and root developmental processes induced upon feeding cell formation on the other hand.

## Data Availability Statement

The datasets presented in this study can be found in online repositories. The names of the repository/repositories and accession number(s) can be found in the article/Supplementary Material.

## Author Contributions

QZ and AG designed the experiment. QZ, ABe, ABr, and CS performed the experiments. QZ, ABe, ABr, SR, and MS analyzed the data. QZ drafted the original manuscript, while AG and MS improved the manuscript. All authors read and approved the submitted version.

## Conflict of Interest

The authors declare that the research was conducted in the absence of any commercial or financial relationships that could be construed as a potential conflict of interest.

## Publisher’s Note

All claims expressed in this article are solely those of the authors and do not necessarily represent those of their affiliated organizations, or those of the publisher, the editors and the reviewers. Any product that may be evaluated in this article, or claim that may be made by its manufacturer, is not guaranteed or endorsed by the publisher.

## Footnotes

1
https://biit.cs.ut.ee/gprofiler/gost
