# Supplementary material for: Comparative Transcriptome Analysis Reveals the Specific Activation of Defense Pathways Against Globodera pallida in Gpa2 Resistant Potato Roots
Source: Front Plant Sci. 2022 Jun 17;13:909593. doi: 10.3389/fpls.2022.909593 (PMC9248836; doi:10.3389/fpls.2022.909593)
Supplement: Supplementary file 1 [file Data_Sheet_1.docx]

# Supplementary Material

**
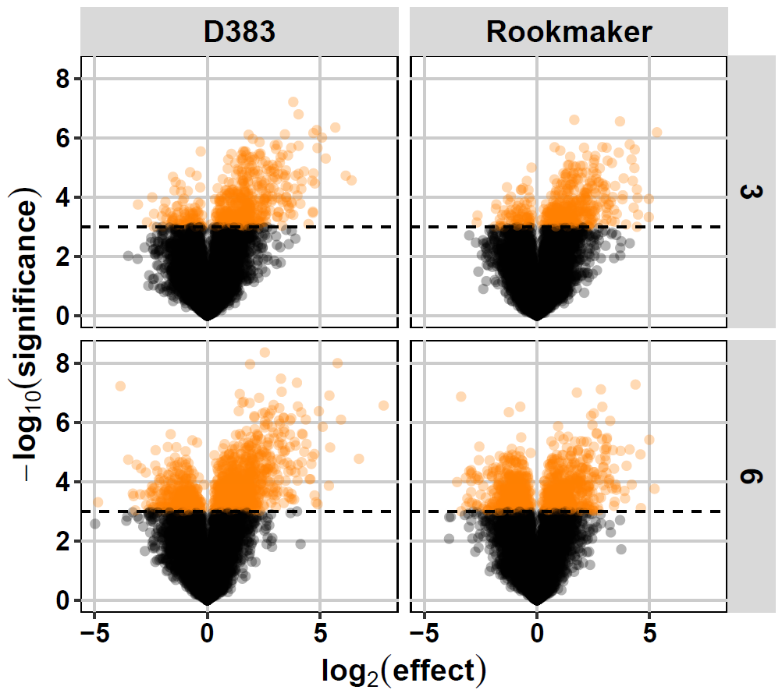
**

**Supplementary Figure 1.** Volcano plot of differentially regulated genes affected by *G. pallida* infection at 3 and 6 dpi. Differentially expressed genes were labelled as black dots. The gene expression change with the threshold of false discovery rate (fdr < 0.05) were labelled as orange dots.


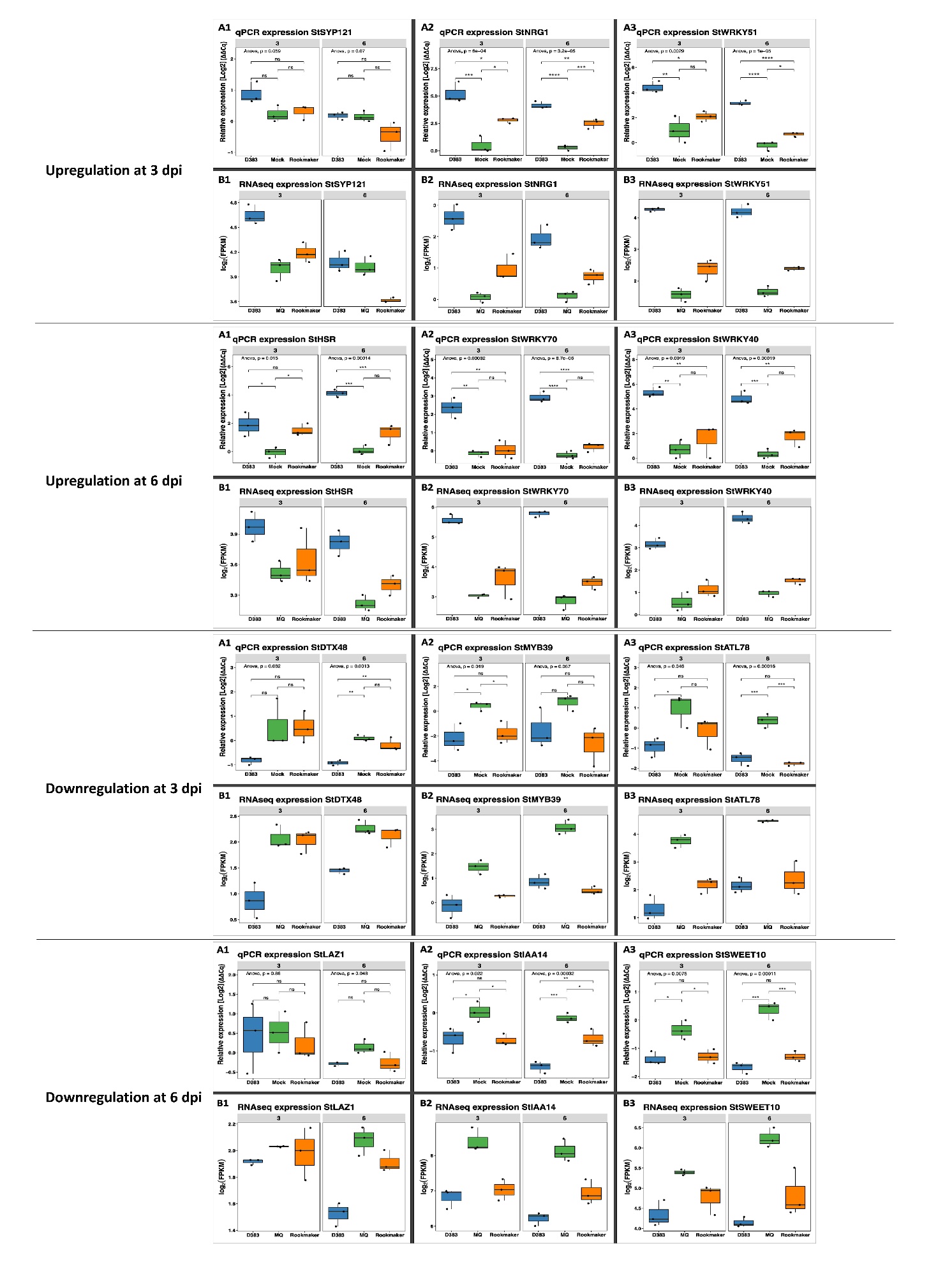


**Supplementary Figure 2. Validation of the expression levels of some plant defense related differentially expressed genes (DEGs). A)** Boxplots of qRT-PCR analysis of the target genes upon *G. pallida* infection. The transcript expression is shown in normalized relative expression (ΔΔCq) logarithmically transformed (Log_2_). The data of these genes was normalized using two reference genes. StSYP121 (PGSC0003DMG400021331) and StWRKY51 (PGSC0003DMG400031140) were normalized to StTUA5 and StMST2. StNRG1 (PGSC0003DMG400010226), StHSR (PGSC0003DMG400022574), StWRKY70 (PGSC0003DMG400020608), StWRKY40 (PGSC0003DMG400007387), StDTX48 (PGSC0003DMG400006378), StMYB39 (PGSC0003DMG400026628) and StLAZ1 (PGSC0003DMG400016039) were normalized to StOXA1 and StMST2. StATL78 (PGSC0003DMG400006044), StIAA14 (PGSC0003DMG400006093) and StSWEET10 (PGSC0003DMG401031741) were normalized to StRPN7 and StTUA5. P-values of the one-way ANOVA comparison of qRT-PCR data are indicated above the boxplot. TukeyHSD pairwise comparison is indicated by the by stars above the brackets spanning the involved treatments. The significance of the p-values = 0.0001, ‘****’ 0.001, ‘***’ 0.01, ‘**’ 0.05, ‘*’, no significance = ‘ns’ **B)** Boxplots of RNAseq analysis of the DEGs upon *G. pallida* infection. Transcript expression is shown in Log_2_ transformed FPKM (Fragments Per Kilobase of transcript per Mapped fragments).


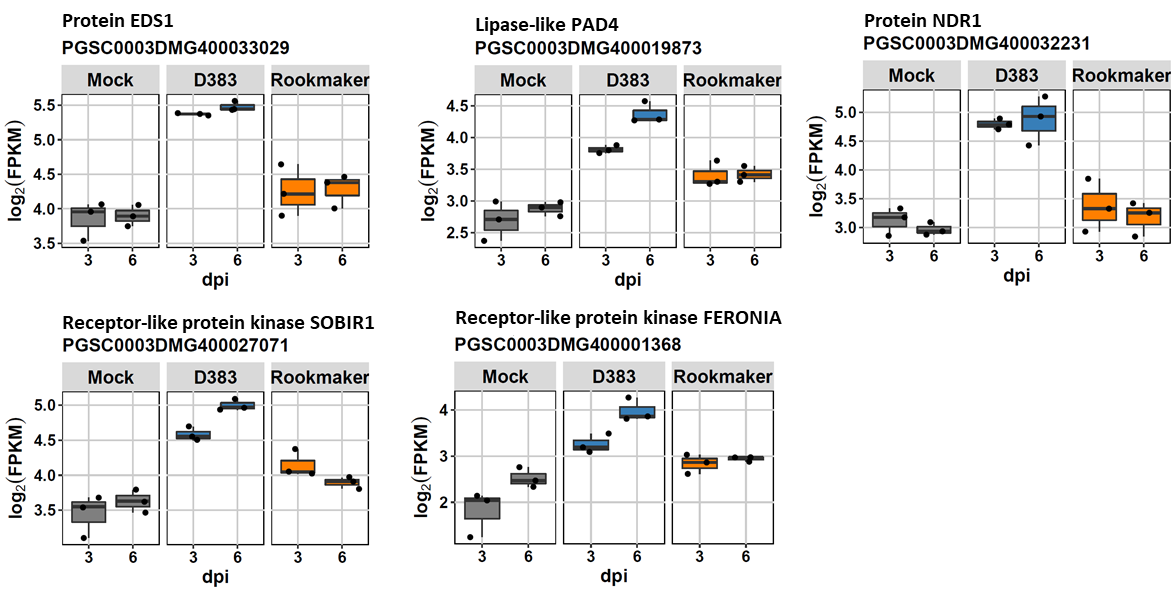


**Supplementary Figure 3.** Expression level of representative D383-specific DEGs (log_2_<2) that relate to plant defense response.

**Supplementary Table 1. Primer sequences used for qRT-PCR analysis.** Primers were designed using NCBI primer BLAST.

**Supplementary Table 2. Detailed information of transcriptome sequencing.**

**Supplementary Table 3. List of DEGs (p < 0.001, fdr < 0.05) from the linear model.**

**Supplementary Table 4. List of DEGs (p < 0.001, fdr < 0.05) enriched in Venn diagram.** The log_2_ (folder change) values are listed.

**Supplementary Table 5. K-means clustering analysis of DEGs from Venn diagram.** The DEGs are divided into 5 clusters.

**Supplementary Table 6. List of DEGs (log_2_>2) specifically respond to D383 infection at 3 dpi, 6 dpi or both 3 and 6 dpi.** The log_2_ (folder change) values in D383 group and k-means cluster are listed.

Supplementary Table 1-6 see separate excel documents.
